# The Keystone Veterinary Medical Association

**Published:** 1901-08

**Authors:** E. M. Ranck

**Affiliations:** Secretary


### THE KEYSTONE VETERINARY MEDICAL ASSOCIATION.

### MAY.

THE regular monthly meeting of the Keystone Veterinary Medical Association was held in the regular meeting place on May 14th, with the following members present: Drs. Harger, Goentner, Lintz, Huidekoper, J. C. Morris, M.D., Hoskins, Rhoads, Ferley, Carter, Marshall and Ranck.

Dr. Carter read a very excellent paper on tumors, which was enjoyed by all, refreshing our memories on the technical points which one is so apt to forget. The discussion was entered into by all present, many questions asked and answered, many cases of tumors cited, some of which were extremely difficult to diagnose. Dr. Carter also presented specimens of a few different tumors for illustration.

A vote of thanks was extended the Doctor for his carefully prepared paper. After the discussion the meeting adjourned for lunch.

E. M. RANCK,
Secretary.

### JUNE.

THE regular monthly meeting of the Keystone Veterinary Medical Association was held on June 11th, 1901, at N. W. cor. Broad and Filbert Streets, with the following members present: Drs. W. H. Hoskins, J. B. Rayner, C. J. Marshall, J. M. Carter, W. L. Rhoads, R. S. Huidekoper, M. W. Drake, Chas. Lintz, R. L. Kann, and E. M. Ranck.

The minutes of previous meeting were read and approved.

The President appointed a committee of five to go to Atlantic City on Sunday next for the purpose of making arrangements to co-operate with the committee of the A. V. M. A. for the annual meeting in September.

The following resolutions were passed: That it is the sense of this Association that clinics be dispensed with at the A. V. M. A. meeting.

That if clinics are held, that they be held on the day following the regular days of meeting of the A. V. M. A., and that the committee appointed at this meeting should confer with the committee of A. V. M. A. in the hope of having this brought about. Carried.

A short address was made by Dr. R. S. Huidekoper on wounds and traumatism in the region of metacarpal and metatarsal phalangeal articulations. The Doctor dwelt to a great degree on the anatomy of the ligaments and synovials, outlining in each instance prognosis, complications, variations and treatment. A plaster cast model was used by which the details of this very interesting subject were demonstrated. The subject was thoroughly discussed, and all felt the benefit of hearing the whole field rehearsed in such a simple manner.

Dr. Rhoads read a partial history of this Association, in which he reports the fact that a regular meeting of this Association was held on every scheduled meeting night since it was organized. He promises a more complete and thorough report at some future time.

Dr. Raynor reported an interesting case of supposed rabies, showing specimen of brain which he forwarded to laboratory of State Live-stock Sanitary Board for histological examination.

Lunch was served at 10.30 P. M., after which the meeting adjourned to meet September 10, 1901.

E. M. RANCK,
Secretary.

